# Trough concentration may not be a good target for polymyxin B therapeutic drug monitoring

**DOI:** 10.1186/s13054-023-04326-8

**Published:** 2023-01-25

**Authors:** Yuhua Zhao, Huadong Chen, Zhenwei Yu

**Affiliations:** 1 Affiliated Xiaoshan Hospital, Hangzhou Normal University, Hanghzou, China; 2 Affiliated Dongyang Hospital of Wenzhou Medical University, Dongyang, China; 3 Sir Run Run Shaw Hospital, School of Medicine, Zhejiang University, Hangzhou, China

Dear Editor,

Yang et al. published an important study that provided high-quality evidence for the therapeutic drug monitoring (TDM) range of polymyxin B in critically ill patients [1]. We want to add some comments.

It is well known that area under the curve/minimum inhibitory concentration (AUC/MIC) is the index that relates to polymyxin B’s efficacy [2]. Yang et al. found that the polymyxin B AUC_ss,24 h_ threshold of 50–100 mg·h/L was a suitable target for critically ill patients. Moreover, Yang et al. found that trough concentration had a good linear relationship with AUC_ss,24 h_ and trough concentration 1.2–2.8 mg/L could be an alternative for AUC_ss,24 h_ 50–100 mg·h/L in TDM. It is challenging to estimate AUC in routine clinical practice, while trough concentration-only monitoring is much easier and more feasible. However, the R2 of the correlation was only 0.793, and interindividual variance was not neglectable, indicating that the trough concentration could not reflect AUC well for individual patients.

Thus, we performed a Monte Carlo simulation based on a published population pharmacokinetic model, which was developed by Yang’s team and was used for AUC estimation in Yang’s study [3]. NONMEM (version 7.5.0, ICON, Ellicott City, MD, United States) coupled with PDxPop (version 5.3, ICON, Gaithersburg, MD, United States) were used for simulation. Renal clearance was set to 50, 100 and 150 mL/min, and the daily dose was 100, 150 and 200 mg, which was divided into 2 doses. A total of 9000 virtual patients were generated (1000 virtual patients for each clearance and daily dose combination), and the plasma concentration after multiple doses was simulated. AUC_ss,24 h_ of virtual patients were calculated using the pkr package (version 0.1.3) in R software (version 4.0.5) with a linear-up and linear-down method.

The relationship between the simulated trough concentrations and AUC_ss,24 h_ is shown in Fig. 1. A, which is similar to Yang et al.’s study. However, for patients with a trough concentration of 1.2–2.8 mg/L, the AUC_ss,24 h_ ranged from 37 to 216 mg·h/L, and only 73.6% were in the range of 50–100 mg·h/L (Fig. 1B). For patients with an AUC_ss,24 h_ of 50–100 mg·h/L, the trough concentration also varied, and only 61.9% of the patients had a trough concentration of 1.2–2.8 mg/L (Fig. 1C). Thus, trough concentration and AUC_ss,24 h_ of polymyxin B are inconsistent. Trough concentration may not be a good target for polymyxin B TDM from pharmacokinetic simulation results. Future studies are needed to provide a suitable strategy for polymyxin B TDM.

**Fig. 1:**
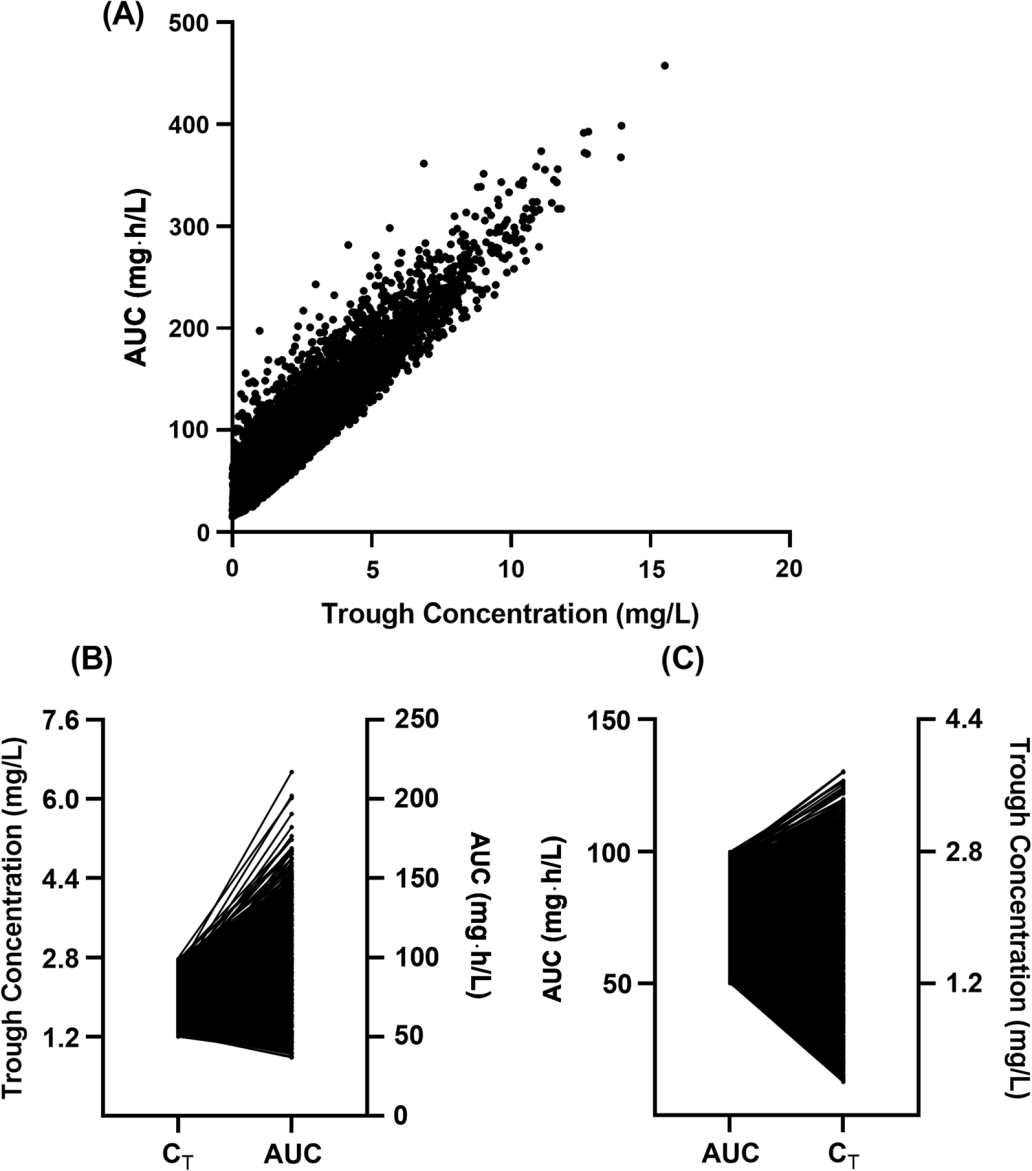
Relationship of trough concentration and area under the curve at steady status after multiple doses of intravenous polymyxin B. AUC: 24-h area under the curve at steady status; C_T_: trough concentration at steady status. **A** Linear relationship of trough concentration and AUC; **B** AUC distribution for patients with trough concentration 1.2–2.8 mg/L; **C** Trough concentration distribution for patients with AUC 50–100 mg·h/L

Yang et al.’s study found a relationship between AUC_ss,24 h_ and clinical response, rather than AUC_ss,24 h_ and mortality. This may be due to the inclusion of patients with various infection sites. The dose of intravenous polymyxin B was suboptimal for lung infection, which was the highest infection rate in the study (305/393) [2, 4]. Plasma concentration or AUC_ss,24 h_ of polymyxin B was also irrelevant to the clinical efficacy of urinary tract infection and cerebral infection, as intravenous polymyxin B was rarely excreted through the kidney or distributed to cerebrospinal fluid [4, 5].

There is an error in Fig. 4(a) of original study that should be addressed. The total number of patients with AUC_ss,24 h_ > 49.1 mg·h/L should be 202, not 393. However, the conclusion was not affected.

## Data Availability

Not applicable.
